# Societal spirits in the silver streak: Unraveling complexity in drinking habits of the mature adult population

**DOI:** 10.1111/acer.15486

**Published:** 2025-01-01

**Authors:** Maarten W. J. van den Ende, René Freichel, Han L. J. van der Maas, Reinout W. Wiers, Sacha Epskamp

**Affiliations:** ^1^ Department of Psychology University of Amsterdam Amsterdam Noord‐Holland The Netherlands; ^2^ Department of Psychology National University of Singapore Singapore Singapore

**Keywords:** addiction, alcohol use, graphical autoregressive modeling, older adult, social network

## Abstract

**Background:**

The complex interactions between an individual's drinking behavior and their social environment is crucial but understudied, particularly in mature adult populations. Our aim is to unravel these complexities by investigating how personal drinking patterns are related to those of one's social environment over time, and what the interplay is with personal factors such as occupational prestige and smoking behavior.

**Method:**

The present study adopts an innovative graphical autoregressive (GVAR) panel network modeling approach to investigate the dynamics between personal drinking habits and social environmental factors, utilizing a comprehensive longitudinal dataset from the Framingham Heart Study with a large sample of predominantly mature adults (*N* = 1719–5718) connected within a social network. We explored both temporal and contemporaneous associations between individuals' drinking habits (self‐reported), smoking behavior (self‐reported), perceived job prestige (Treiman prestige score), and the drinking behaviors of their social environment. The latter consists of the proportion of abstaining, moderate drinking, and heavy drinking social connections of each subject.

**Results:**

Our findings reveal significant associations between participants' behavior and that of their peers, with reciprocal interactions, substantiating the importance of the influence of one's social network for mature individuals. We found dynamic, reciprocal associations between an individual's drinking behavior and that of their peers, with periods of increased or decreased drinking correlating with increased connections to heavy drinkers or abstainers, respectively. In addition, when individuals drink more than usual, they also tend to consume more cigarettes, and vice versa.

**Conclusions:**

The reciprocal feedback loops identified between an individual's drinking behavior and their social environment highlight the crucial role of social influences in shaping drinking behavior, including among older people. This emphasizes the need to consider social elements in the development of future theories, models, and interventions aimed at addressing problematic alcohol consumption in this vulnerable population.

## Graphical abstract

The study utilized Graphical VAR network models on the longitudinal Framingham Heart Study data to examine the associations between individual factors and social environments in 1719–5718 individuals. It was found that drinking behaviors are significantly influenced by peers, both for reducing and increasing drinking. It revealed reciprocal effects on drinking of social connections. The findings underscore the crucial and complex role of social environments in shaping drinking behaviors. The limitations include self‐reported data and incomplete social networks.

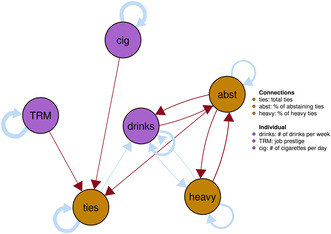

## INTRODUCTION

Substance use disorders are a significant public health problem that affects various demographic groups, spanning different age groups, socioeconomic statuses, and geographical regions (Sudhinaraset et al., 2016). In particular, alcohol use disorder is a widespread and pervasive problem, with effects that extend to both economic and personal levels (Bobo & Husten, 2000), and heavy drinking imposes a high burden on society (Effertz & Mann, 2013; Rehm et al., 2013). Substance use disorders are complex phenomena, with a wide range of influencing factors, ranging from genetic vulnerability to cultural norms, upbringing and the drinking behavior of individuals in their social environment (Brooks‐Russell et al., 2014; Cruz et al., 2012). Research suggests that individuals with higher socioeconomic status tend to consume more alcohol overall but may be less likely to engage in binge drinking, exemplifying the intricate interplay between alcohol consumption and socioeconomic factors (Collins, 2016; Huckle et al., 2010). Use of alternate substances such as tobacco is closely linked to alcohol use and is often seen as a complementary behavior (Bien & Burge, 1990; Blok et al., 2017; Bobo & Husten, 2000; Room, 2004). The two behaviors influence each other at all stages of use; in particular, smoking appears to contribute to alcohol relapse rates, and simultaneous cessation of both habits leads to lower relapse rates (Cooney et al., 2015; Weinberger et al., 2015).

One factor that has a major impact on substance use at all use stages is one's social environment (de Visser, 2021; Sönmez Güngör et al., 2021). It is in and of itself the result of complex dynamics. Factors such as peer pressure, family dynamics, and societal influences play pivotal roles in the initiation and continuation of substance use during adolescence (Donovan, 2004; Hawkins et al., 1992). These influences can have lasting effects on patterns of use into adulthood (Chassin et al., 2002). The influence of community context and family structure is significant, shaping individual behavior and attitudes toward substances even late in life (Moos, Brennan, et al., 2004). Furthermore, peer influences and social drinking motives continue to play a strong role in driving substance use (Freichel et al., 2023). For example, in the same dataset used in the current study, it was found that individuals are 50% more likely to drink heavily if they are connected with heavy drinkers (Rosenquist et al., 2010). The reciprocal nature of these relationships often leads to a feedback loop in which substance use is normalized and reinforced within social circles (Borsari & Carey, 2001). Social support also plays a crucial role in moderating substance use, particularly in terms of recovery and prevention. Friends who abstain from substance use can be a positive influence, reinforcing abstinence and promoting healthier behaviors (Witkiewitz & Marlatt, 2009); in the dataset used in the current study, it was found that being surrounded by abstainers would decrease consumption of individuals by 50% (Rosenquist et al., 2010). Interventions that leverage support from social networks have shown promise in reducing substance use and preventing relapse (Ariss & Fairbairn, 2020; Bliuc et al., 2018; van den Ende et al., 2024). Structured settings such as Alcoholics Anonymous meetings provide supportive communities that can help individuals maintain sobriety, particularly effective for individuals with many heavy drinking connections (Kelly et al., 2011; PMR Group, 1998; Witkiewitz et al., 2007).

Current research on social dynamics and drinking behavior has predominantly focused on adolescents and young adults (Donovan, 2004; Freichel et al., 2023; Hawkins et al., 1992). However, there are compelling reasons to suspect that the social network dynamics observed in younger cohorts may not be applicable to older demographics. For example, while peer influence may decline in later life, other aspects of the social environment, such as the effects of loneliness, may become more dominant (Wrzus et al., 2013). In addition, as people age, continued substance use is often associated with stable coping mechanisms or long‐established habits (Chernick & Kuerbis, 2016; Moos, Schutte, et al., 2004; Simoni‐Wastila & Yang, 2006). At the same time, the physiological effects of alcohol consumption are changing, with alcohol‐related health risks increasing with age (Knox et al., 2019). Despite these known factors, the impact of social networks on alcohol consumption in mature adult populations remains poorly understood and has been identified as an area for further research (Knox et al., 2019). Thus, although the importance of the role of the social environment is undeniable, the dynamics of these complex interactions remain poorly understood, particularly for mature adults. As several reviews indicated, psychological theories and formal modeling efforts often overlook the reciprocal nature of the relationships between individuals and their social environments (Kato et al., 2022; van den Ende et al., 2022; van der Wal et al., 2021). Without a full understanding of this key factor, we may miss potential intervention strategies that are critical for both onset and relapse prevention (Marlatt et al., 2004).

Our research aims to fill the existing gap in understanding the complex relationships between alcohol consumption, personal factors such as tobacco use and job prestige; the perceived social status of one's occupation—a key aspect of socioeconomic status (SES) (Hughes et al., 2022), which we use as an operationalization of SES—and the influence of drinking habits within one's social circle. To achieve this, we use the comprehensive longitudinal social network dataset with participants having an average age of about 55 years. By classifying the drinking behavior of individuals' social connections into abstainers, moderate, and heavy drinkers, we are able to distinguish their different social influences.

Our study uses novel dynamic network modeling techniques to investigate two principal categories of effects: contemporaneous effects and temporal effects (Epskamp, 2020). The former covers the simultaneous associations that occur within the same time frame, aiming to elucidate how various variables might be interrelated at a specific moment. In contrast, the latter focuses on the average effects that unfold over time within an individual, shedding light on the progression of certain factors and their impact on an individual's behavior or experiences. A critical element of the research is the differentiation between within‐person and between‐person effects (Curran & Bauer, 2011). Within‐person effects examine the changes occurring in individuals over time, whereas between‐person effects analyze the persistent differences among various individuals. The distinction between these effects is crucial; interventions designed to modify social network dynamics must be informed by a nuanced understanding of these individual processes.

## METHODS

### Data source

Our analysis uses data from the Framingham Heart Study [dataset] (Dawber et al., 1951), an ongoing longitudinal cohort study initiated in 1948. The data are derived from two cohorts:

1. The “Original Cohort” of 5209 initial participants, started in 1948. Mean age between 63.9 (wave 1) and 80.7 (wave 5).
2. The “Offspring Cohort” of 5124 initial participants, started in 1971. Mean age: 36.7 (wave 1) and 58.2 (wave 7).

The dataset provides an extensive array of information collected over decades, primarily centered on physical health obtained from clinical examinations. Included within this dataset are a limited number of lifestyle variables that are relevant to our research, notably alcohol consumption, cigarette use and a measure of job prestige. It also contains the “Social Net”, a social network constructed from participants' reported relationships and additional data such as family ties and address records (Christakis & Fowler, 2007). By aligning the annual data from the Original Cohort with the nearest corresponding data from the Offspring Cohort, we have aggregated the data into nearly 3‐year intervals, spanning seven waves of assessment over a 32‐year period between 1971 and 2003.

### Individual measures

We included individual behavioral measures focused on drinking habits, smoking behaviors, and occupational prestige. Drinking behavior is determined by self‐reporting the average number of drinks consumed per week in the past year, asking separately for beer, wine, and liquor. We summed these different types to obtain a total drink count for every participant. Similarly, the item “cigarettes per day” assessed the self‐reported average number of cigarettes smoked per day in the previous year.

Social status was assessed using the Treiman occupational prestige score (Treiman, 2013). Occupations receive a score following a survey in which respondents rate the prestige of various occupations. Higher scores correlate with greater prestige. This measure does not correspond exactly to SES (Socio‐economic Status), which includes more than just occupation, but it can serve as an indicative measure of social status.

### Social measures

Our social network data includes three primary metrics. The first metric, Total Connections, represents the total number of social connections recorded in the dataset for each participant. These connections consist of a small number of self‐reported friendships, recorded various familial ties, and individuals living at the same address. For our analysis of social connections, we excluded co‐workers and spatial neighbors, as there is insufficient evidence that these are actual social connections (Christakis & Fowler, 2008) or whether these types of connections significantly influence each other's drinking behavior (Rosenquist et al., 2010). We also excluded individuals with no or only one known connection, as this removes individuals for whom the social network data is severely lacking which makes their social environment not suitable for an analysis that categorizes connections into proportions based on the drinking status.

Second, to assess social drinking environment, we have classified the connections into three categories based on their drinking habits. This categorization, as proposed by Epskamp et al. (2022) and confirmed as a valid option in the FHS by van den Ende et al. (2024), consists of abstainers (0 weekly consumptions), moderate drinkers (1–7 for women, 1–14 for men (Gunzerath et al., 2004), and heavy drinkers (>7 for women, >14 for men)). Given that the total number of connections varies for each individual, the number of social connections in each category does not serve as a direct indicator of the social drinking environment. Instead, it is more closely related to the total number of connections. To obtain a more accurate representation of the drinking environment, we calculate for each individual the relative proportion of connections in each of the three drinking categories. We then use the proportion of abstainers and of heavy drinkers as variables, indicating to what extent that one's social environment lies outside of the norm of moderate drinkers. We also include the total number of ties, which we expect to take into account the longitudinal effects of data attrition, as the reduction in the number of ties is mostly due to death.

### Data analysis

A panel graphical vector autoregression model (GVAR) (Epskamp et al., 2020) was used to model the interaction between individual factors (number of drinks, cigarettes, job prestige) and social connections. The panel GVAR model is similar to a random intercept cross‐lagged panel model (Epskamp, 2020). We used full information maximum likelihood (FIML) estimation; a preeminent approach minimizing bias in the estimates similar to multiple imputation procedures, which assumes data missing at random. The model estimates autoregressive (i.e. factors, represented as nodes, predict themselves) and cross‐lagged (i.e. different nodes predict each other) associations. The innovation (co‐) variances of the model are modeled by a Gaussian graphical model (GGM) (Epskamp et al., 2018), to obtain contemporaneous partial associations (within the same time window). Therefore, the temporal dependencies of the data structure are taken into account. The model was estimated using the psychonetrics R package (Epskamp et al., 2020) and visualized using the qgraph R package (Epskamp et al., 2012). After estimating a comprehensive model that includes all effects, each represented as an edge with its corresponding strength as the edge weight, the model was pruned at an *α* level of 0.05 (Blanken et al., 2022). The pruning process involves removing nonsignificant effects and re‐estimating the model with nonsignificant estimates set to zero. In addition, a sensitivity analysis was conducted using solely the Offspring Cohort, using the same methods.

## RESULTS

As shown in Table 1, describing the sample characteristics per wave, the initial sample consisted of *n* = 5718 individuals. There was substantial attrition with *n* = 1719 individuals at the final wave. This attrition was mainly due to the death of members of the original cohort as they aged. Attrition analysis, detailed in Table S1, indicates that participants who dropped out were relatively similar to those who completed the study in terms social connections and alcohol consumption. However, cigarette usage was significantly higher among the participants who dropped out, with an average increase of 45%, indicative of the lethal effect of smoking. In addition, job prestige score was significantly lower among participants who dropped out at 43.80 compared to 46.26.

**TABLE 1:** Sample characteristics of all examination waves.

Wave	*N*	Mean (std)	Mean (std)	Men	Mean (std)	% heavy	%
		Drinks	Age	%	Contacts	Drinkers	Abstain
1	5718	7.55 (10.74)	47.98 (15.97)	53.28	4.13 (1.81)	22.35	19.81
2	3962	7.12 (10.48)	53.32 (16.38)	54.18	3.78 (1.68)	21.31	31.42
3	3355	6.29 (9.34)	55.17 (15.51)	54.84	3.65 (1.68)	18.44	35.30
4	3244	5.34 (8.56)	57.20 (15.17)	53.83	3.62 (1.85)	14.83	38.12
5	2810	4.96 (7.75)	59.41 (14.28)	54.80	3.50 (1.87)	14.00	38.07
6	1682	5.12 (8.01)	58.19 (9.65)	51.91	3.01 (1.29)	14.97	40.31
7	1719	5.42 (8.23)	60.82 (9.60)	52.32	2.99 (1.32)	16.25	35.71

*Note*: Columns “% Heavy Drinkers” and “% Abstain” show the drinking behavior of the social connections; the mean of the percentage of connections of all individuals that drink heavily or abstain respectively.

The average number of connections saw a gradual decrease from 4.03 to 2.99. In terms of alcohol consumption, the average number of drinks consumed per week started at 7.62, but fell to around five in subsequent waves. The standard deviation for this metric ranged from 7.7 to 10.7. Over the 32 years of the study, the average age of participants increased from 48.0 to 60.8. The average number of abstaining contacts varied between 0.75 and 1.23, with most waves approximating 1.10. The average number of heavy drinking connections showed a slight decline from 0.90 in the first wave, to about 0.65 in the later waves. We applied the saturated panel network model to our data and obtained an excellent fit (RMSEA = 0.039, CFI = 0.92, TLI = 0.92) (Du et al., 2024; Epskamp et al., 2020). To increase robustness against false positive findings, we implemented standard pruning procedures with *α* = 0.05. This resulted in a similar good fit of (RMSEA = 0.038, CFI = 0.92, TLI = 0.92).

Furthermore, the results of the analysis utilizing only the Offspring Cohort are found in Figures S1, S2, and S3. This approach negates nesting within families, albeit with slightly reduced statistical power (RMSEA = 0.045, CFI = 0.92, TLI = 0.92).

### Contemporaneous network

**FIGURE 1:**
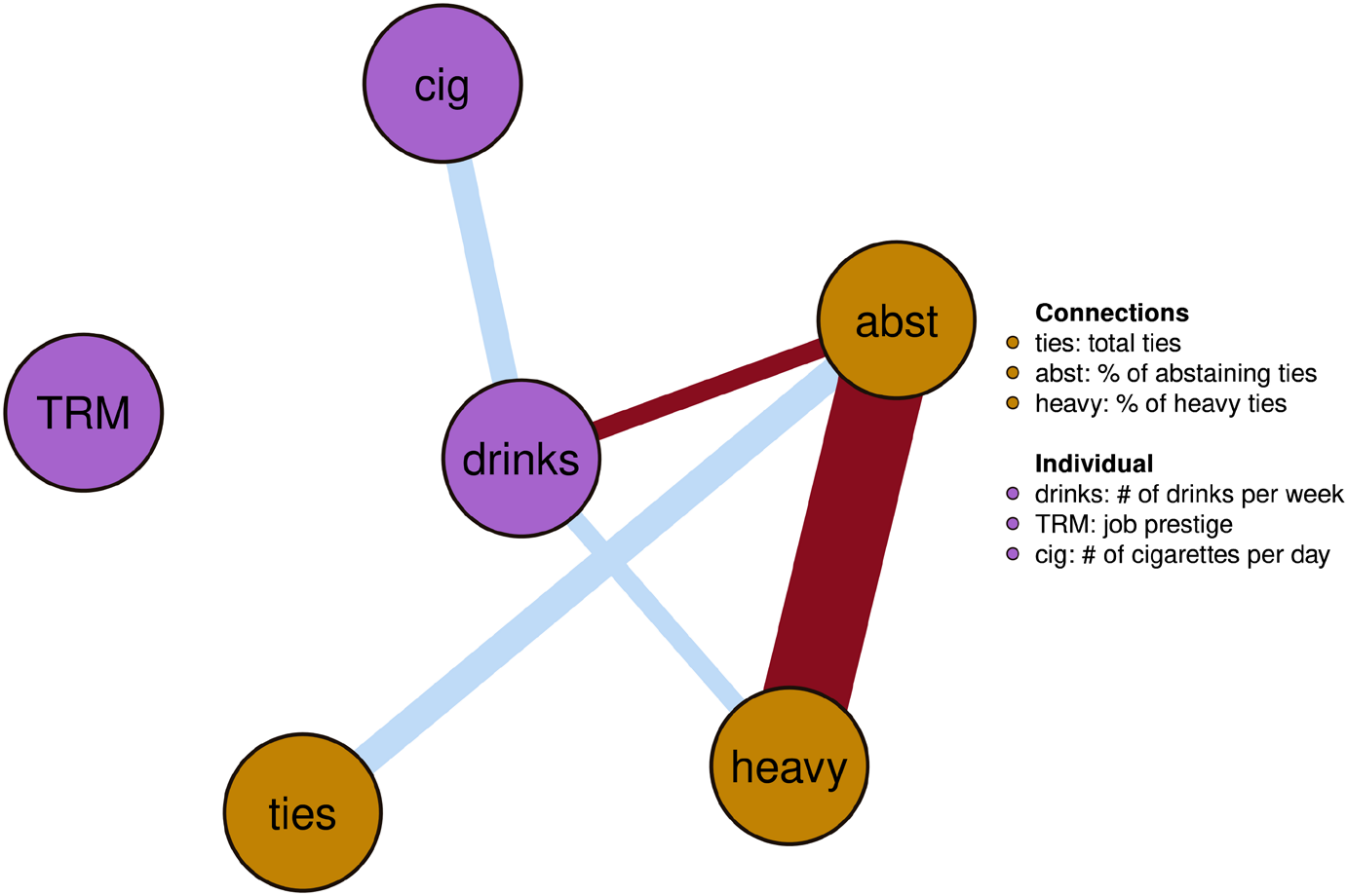
Fixed‐effect contemporaneous associations within the same time window. Blue are positive and red are negative associations, the width of the edge represents the strength. Node color indicates personal (purple) or social (gold) variable.

The contemporaneous network, shown in Figure 1, displays the partial correlations among all variables at a given measurement point, while adjusting for their time‐related interdependencies (Epskamp, 2020). This illustrates the (fixed‐effect) influences of different behaviors and social environment within individuals at that particular time.

There is a large negative association between abstaining connections and heavy drinking connections, which is an expected result of our operationalization of connections: any abstaining connection is by definition not a heavy drinking connection and therefore always reduces the proportion of heavy drinking connections. Therefore, this multicollinearity effect is not necessarily indicative of any impact. Its edge weight of −0.22 is small enough to not impact other findings.

The network reveals strong association between drinking behavior and the social environment. Notably, the quantity of alcohol consumed appears to co‐occur positively with social connections that frequently engage in heavy drinking and negatively co‐occur with abstaining connections. This pattern might suggest that when participants drink more than they usually do, they could potentially gain more heavy drinking connections. The network also highlights a strong positive co‐occurrence of alcohol and cigarette use. This association indicates that individuals who consume more alcohol than they typically would also tend to smoke more frequently, and vice versa.

### Between‐subjects network

**FIGURE 2:**
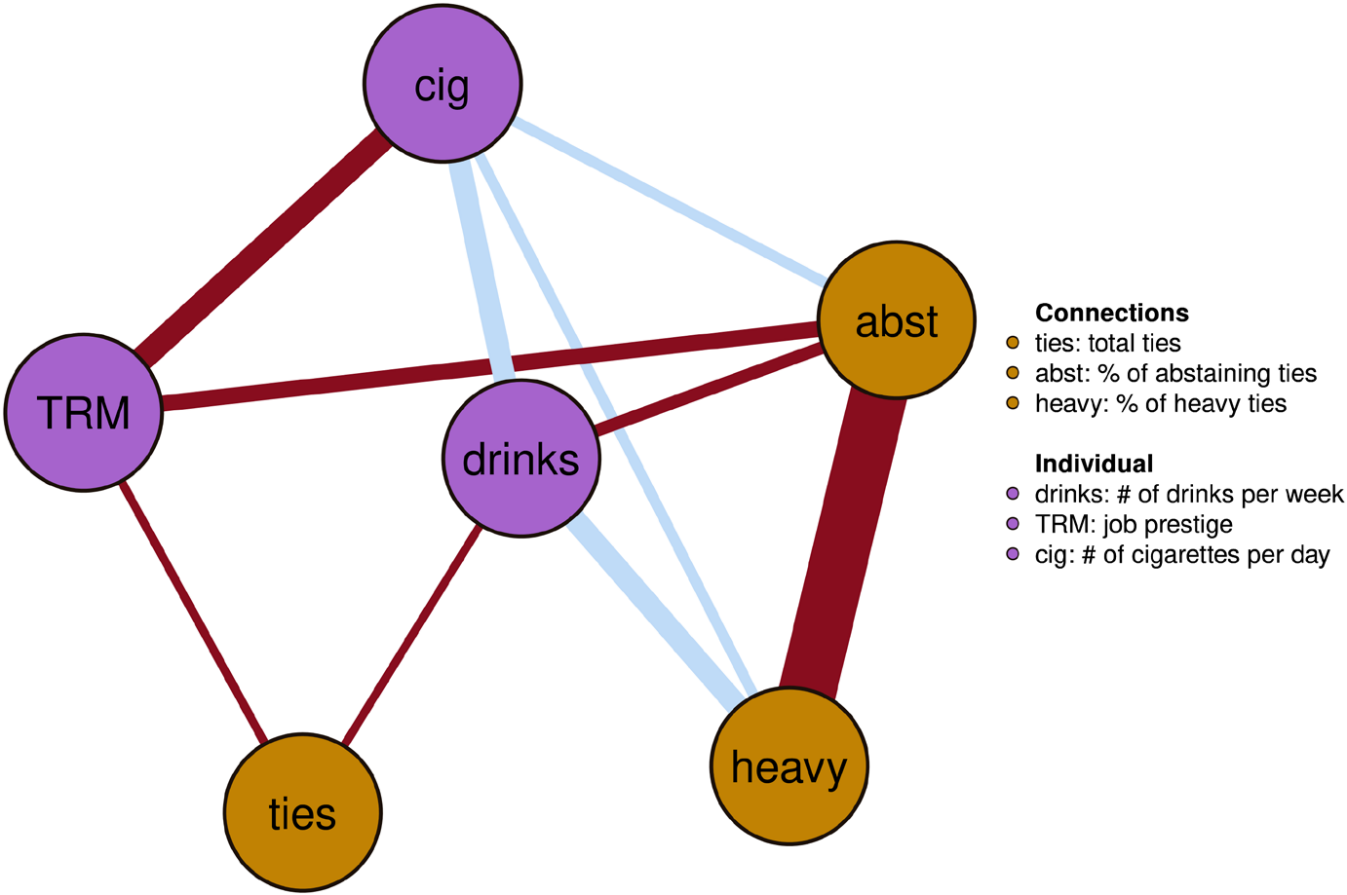
Fixed‐effect between associations within the same time window. Blue are positive and red are negative associations, the width of the edge represents the strength. Node color indicates personal (purple) or social (gold) variable.

The between‐subjects network, depicted in Figure 2, represents an undirected partial correlation network that captures the relationships between individuals' consistent average behaviors; this reflects the typical patterns observed across the entire population. We observed a notable association between social environment and drinking behavior. Individuals who typically consume more alcohol seem to have fewer abstaining connections and more heavy drinking connections. Conversely, people with more heavy drinking connections generally appear to consume more alcohol themselves. We also found a positive association between smoking and drinking. This suggests that people who tend to smoke more cigarettes also seem to consume more alcohol, and vice versa. Interestingly, our data show that individuals who smoke more often have a higher percentage of abstaining connections as well as connections to heavy drinkers, implying that smokers may have fewer moderate drinking connections. Lastly, the network suggests that individuals with higher job prestige often have fewer abstaining connections and tend to smoke less on average.

### Temporal network

**FIGURE 3:**
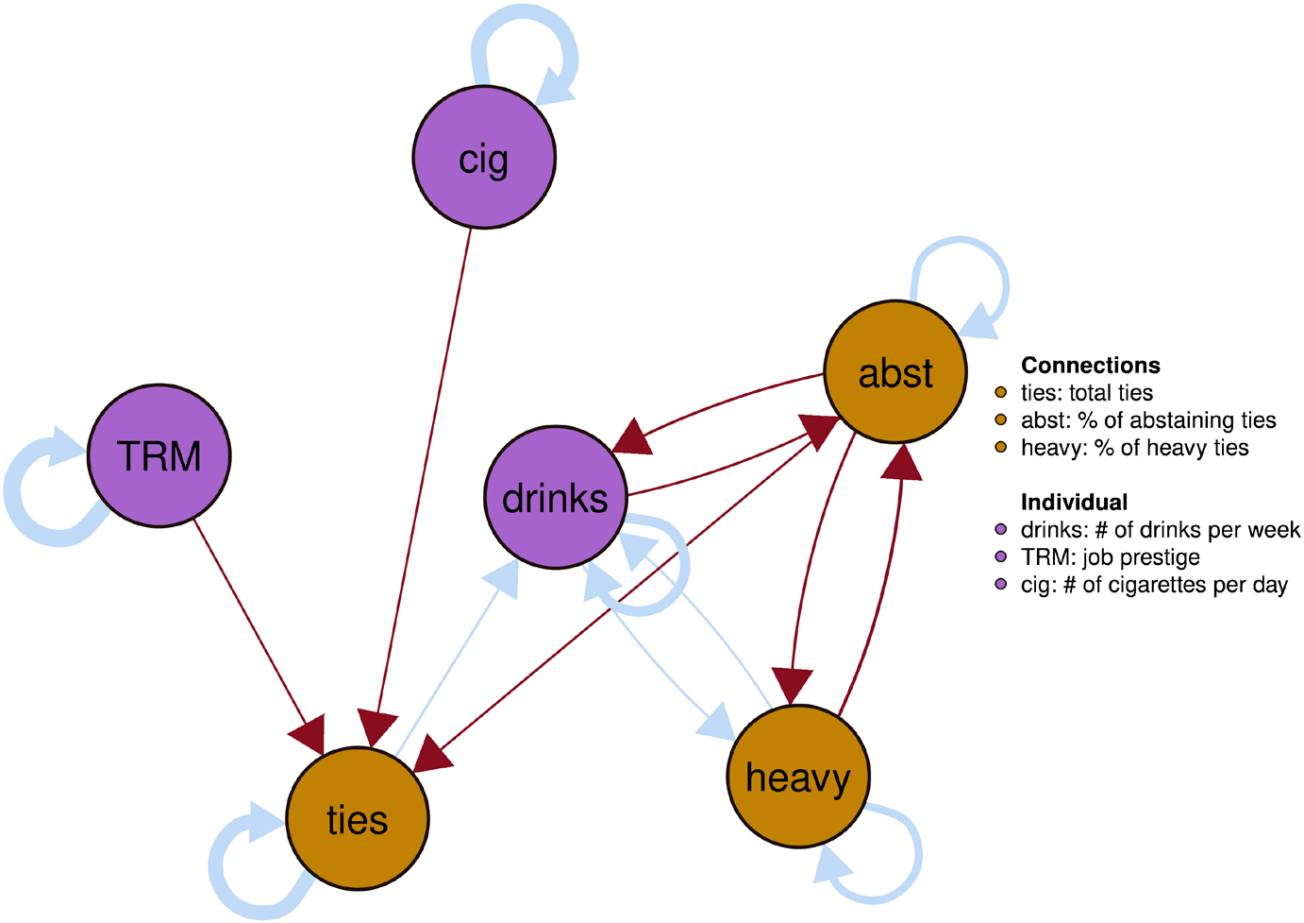
Fixed effect temporal associations. Blue are positive and red are negative associations. The thickness of the arrow indicates the strength of the association. Node color indicates personal (purple) or social (gold) drinking behavior variables.

The temporal network depicted in Figure 3 illustrates potential predictive associations between variables (i.e., partial directed correlations) between an individual's social drinking environment (gold) and measures of alcohol use, cigarette use, and job prestige (purple). This within‐person level network yields a pattern characterized by bidirectional feedback loops and autoregressive effects (i.e., a node predicting itself). These patterns may suggest a reciprocal influence between an individual's drinking behavior and their social environment's drinking habits. For instance, alterations in an individual's drinking habits might correspond with a change in their social connections with other drinkers. Similarly, changes in one's social connections could potentially influence their own drinking behavior; the total number of social connections appears to predict an increase in drinking behavior. However, variations in job prestige or smoking behavior do not seem to impact drinking behavior according to this analysis.

## DISCUSSION

In the present study, we used an innovative analysis, based on Graphical VAR network models, to investigate the associations between individual and social environmental factors. We leveraged the comprehensive longitudinal dataset from the Framingham Heart Study, including its social network data, to distinguish between within‐person and between‐person associations in a sample of consisting of between *N* = 5718 and *N* = 1719 individuals. In addition, we modeled temporal and contemporaneous relationships between an individual's drinking habits, smoking behavior, perceived job prestige and, notably, the drinking behaviors of their social connections. This approach is notable for its innovative consideration of an individual's social network in relation to their personal habits, as well as the novel application of GVAR to factors that extend beyond personal symptoms or traits. Hence, we were able to explore not only the correlations between individuals but also the correlations within individuals and the unique dynamics of how these factors influence other factors over time.

At the contemporaneous level, we find that drinking behavior is similar to the drinking behavior of peers. It also confirms that smoking and drinking often go together (Bobo & Husten, 2000; Room, 2004); if one drinks more than usual, one also tends to consume more cigarettes. These findings are consistent with the between‐subjects network that also shows a negative association between job prestige scores and cigarette use and alcohol abstinence, supporting prior research that higher socio‐economic status tends to be associated with more regular drinking but less smoking (Hiscock et al., 2012; Huckle et al., 2010). Note that the high multicollinearity between alcohol abstinence and heavy drinking connections, an inherent result of using the proportion of connections, might mask their distinct associations with other variables and reduce the likelihood of detecting different effects. We expect this not to be the case however, as edges with different nodes still persist, and the actual edge weight of −0.22 is smaller than the visualization might imply.

The temporal network revealed dynamic associations between an individual's factors and their peers' drinking behavior. We find positive feedback loops in the spread of drinking behavior (induction (Christakis & Fowler, 2007)); our analysis suggests that drinking more over time also increases one's proportion of heavy drinking connections, and drinking less increases ones abstaining connections over time. An increase in the number of heavy drinking connections results in an increase in drinking. However, this edge did not replicate when we conducted a sensitivity analysis with only the Offspring Cohort. Similarly, increasing the number of abstaining connections makes one drink less over time. Again, this could be due to induction, but it could also be due to homophily, a process where changing drinking behavior leads people to change their social connections to better match their drinking behavior. This results in a positive feedback loop, demonstrating the existence of nonlinear dynamics in the interplay between one's drinking behavior and their social environment.

Our results substantiate previous findings that drinking behavior within the same time period is highly correlated with the drinking behavior of peers, even for mature individuals (Borsari & Carey, 2001; Cruz et al., 2012; Larsen et al., 2012; Morris et al., 2020; Sudhinaraset et al., 2016). It also builds on research conducted by Rosenquist et al. (2010) which uses the same dataset. Using bivariate regression, they found that being surrounded by heavy drinkers was associated with an increase in the number of drinks consumed. By being able to distinguish a reciprocal effect, our study offers a more nuanced understanding of the complex nature of interactions within the social environment, and the positive feedback loops found highlight the importance of interventions at the social network level, even for older people.

This finding is reinforced by the absence of clear associations over time between smoking habits, job prestige and drinking, suggesting that the social environment is a more influential factor in modifying drinking behavior than these individual factors. This is the case for increased drinking associated with heavy drinking as well as for abstinent relationships and reduced drinking, corroborating previous research (Kelly et al., 2011; Rees & Wallace, 2015). This finding is consistent with previous research suggesting that fostering supportive social networks can be particularly effective in promoting sobriety, as demonstrated by organizations such as Alcoholics Anonymous (Bond et al., 2003; Groh et al., 2008; Longabaugh et al., 1998).

Our findings need to be interpreted with a number of limitations in mind. First, the dataset used has inherent limitations. Drinking and smoking behaviors were self‐reported, potentially compromising their accuracy (Del Boca & Darkes, 2003).

Second, the social network data are not fully comprehensive. The Framingham Heart Study was not primarily designed to capture a complete social network and is therefore limited in the number of connections, particularly in terms of friendships (Christakis & Fowler, 2007). Furthermore, certain connections, such as family ties, were collected through municipal databases, and we cannot conclusively verify that these are actual social connections. In addition, job prestige might not be a fully accurate operationalization of socio‐economic status, and our measurement only considers quantity of drinking, which may not fully capture the nuances associated with varying frequencies of alcohol consumption. Finally, there is a degree of variability in the time between observations; they range from 2 to 3 years with an outlier of 8 years—while the panel GVAR model assumes an approximately stationary time‐series. Although studies have shown that alcohol consumption remains stable within 2–4 year windows (Epskamp et al., 2022; van den Ende et al., 2024), the extended duration between study waves could mean that short‐term variability may not be captured. In addition, the current panel GVAR model can only uncover linear, lag‐1 associations, meaning it cannot capture nonlinear dynamics or correlations at different time lags (Epskamp et al., 2020).

In addition, while the temporal associations imply Granger causality, suggesting temporal precedence and directional effects that are useful for exploring potential mechanisms and informing hypothesis generation, they do not establish definitive causality. These relationships can, therefore, guide further research but must be interpreted with caution, recognizing that they are indicative rather than confirmatory. Lastly, it is important to note that our dataset, collected between 1971 and 2003, reflects the cultural and social context of that time period. Given potential shifts in culture and drinking behaviors in more recent years, our findings may not be directly applicable to contemporary populations.

Our results highlight the pivotal role of the social environment in shaping individual drinking behavior, apparent in all network models evaluated. In particular, the temporal model revealed bidirectional feedback loops between individuals and their social contexts, highlighting that an individual's drinking behavior is both influenced by and contributes to the dynamics within their social environment. Recognizing the importance of these interactions is not only promising but essential for a comprehensive understanding of drinking behavior. Consequently, we suggest that future research efforts should focus on unraveling the complexity of these social factors. In addition, future research conducting similar analyses with access to more detailed data could aim to examine these dynamics within more specific populations, such as different sexes or age groups. Future theoretical and empirical work would greatly benefit from incorporating social dimensions to better understand and address alcohol consumption patterns and to develop more effective intervention strategies.

## AUTHOR CONTRIBUTIONS

M.W.J. van den Ende analyzed the data. M.W.J. van den Ende, R. Freichel, H.L.J. van der Maas, S. Epskamp, and R.W. Wiers were involved in the conceptualization and writing process. All authors reviewed the manuscript.

## FUNDING INFORMATION

Research within the Centre for Urban Mental Health is funded by the University of Amsterdam. The Framingham Heart Study is conducted and supported by the National Heart, Lung, and Blood Institute (NHLBI) in collaboration with Boston University (Contract Nos. N01‐HC‐25195, HHSN268201500001I and 75N92019D00031). This manuscript was not prepared in collaboration with investigators of the Framingham Heart Study and does not necessarily reflect the opinions or views of the Framingham Heart Study, Boston University, or NHLBI. Funding for SHARe genotyping was provided by NHLBI Contract N02‐HL‐64278. Funding support for the Framingham Social Network datasets was provided by NIA grant P01 AG 031093.

## CONFLICT OF INTEREST STATEMENT

All authors declare that there are no conflicts of interest related to this study. We have no financial or personal connections that could have influenced the research outcomes. Our work remains independent and unbiased.

## Supporting information


Data S1.

## Data Availability

The data, comprising clinical exams and demographic details such as age and sex, are sourced from the Framingham Cohort study (reference: phs000007.v33.p14). The social network information is derived from the FHS‐Net Social Networks substudy (reference: phs000153.v9.p8). The supporting data for this study's findings are accessible via the NCBI database dbGaP. However, due to privacy and ethical restrictions on these data, which were utilized under license for this investigation, they are not publicly accessible. Contact information, details on the data, and instructions for requesting access can be found on the following websites: Framingham Cohort: https://www.ncbi.nlm.nih.gov/projects/gap/cgi‐bin/study.cgi?study_id=phs000007.v33.p14, FHS‐Net Social Networks: https://www.ncbi.nlm.nih.gov/projects/gap/cgi‐bin/study.cgi?study_id=phs000153.v9.p8.
