# Supplementary material for: Societal spirits in the silver streak: Unraveling complexity in drinking habits of the mature adult population
Source: Alcohol Clin Exp Res (Hoboken). 2025 Jan 1;49(1):217–25. doi: 10.1111/acer.15486 (PMC11740162; doi:10.1111/acer.15486)
Supplement: Supplementary file 1 — Data S1. [file ACER-49-217-s001.pdf]

Measure	Completed Mean (SD)	Dropout Mean (SD)	p value
Drinks per week	4.25 (5.12)	4.44 (6.48)	0.37
TRM: Job Prestige	46.26 (11.91)	43.80 (11.88)	> 0.001
Number of cigarettes per day	7.11 (11.41)	10.26 (12.75)	> 0.001
Total number of contacts	3.65 (2.00)	3.75 (1.98)	0.14
Percentage of abstaining ties	0.17 (0.24)	0.19 (0.26)	0.04
Percentage of heavy drinking ties	0.24 (0.28)	0.24 (0.29)	0.50

Table S1: Attrition analysis of all participants in terms of wave 1, comparing participants who completed the study with those lost to attrition using a two-sided t-test.

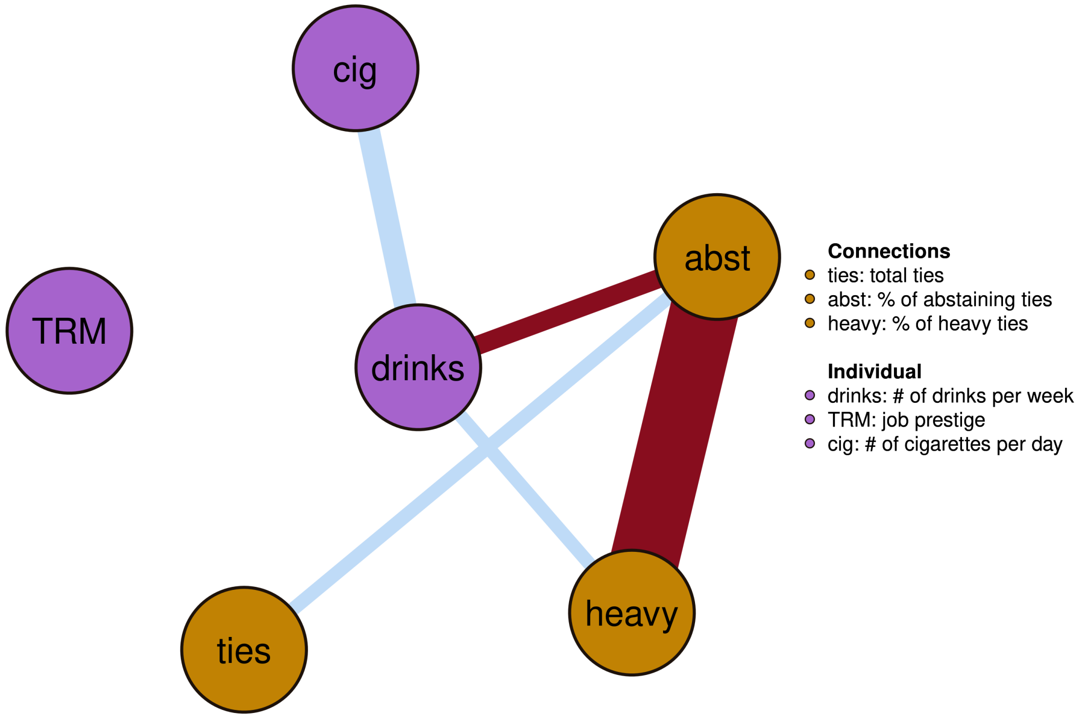

Figure S1: Fixed-effect contemporaneous associations within the same time window of solely the Offspring Cohort. Blue are positive and red are negative associations, the width of the edge represents the strength. Node color indicates personal (purple) or social (gold) variable.

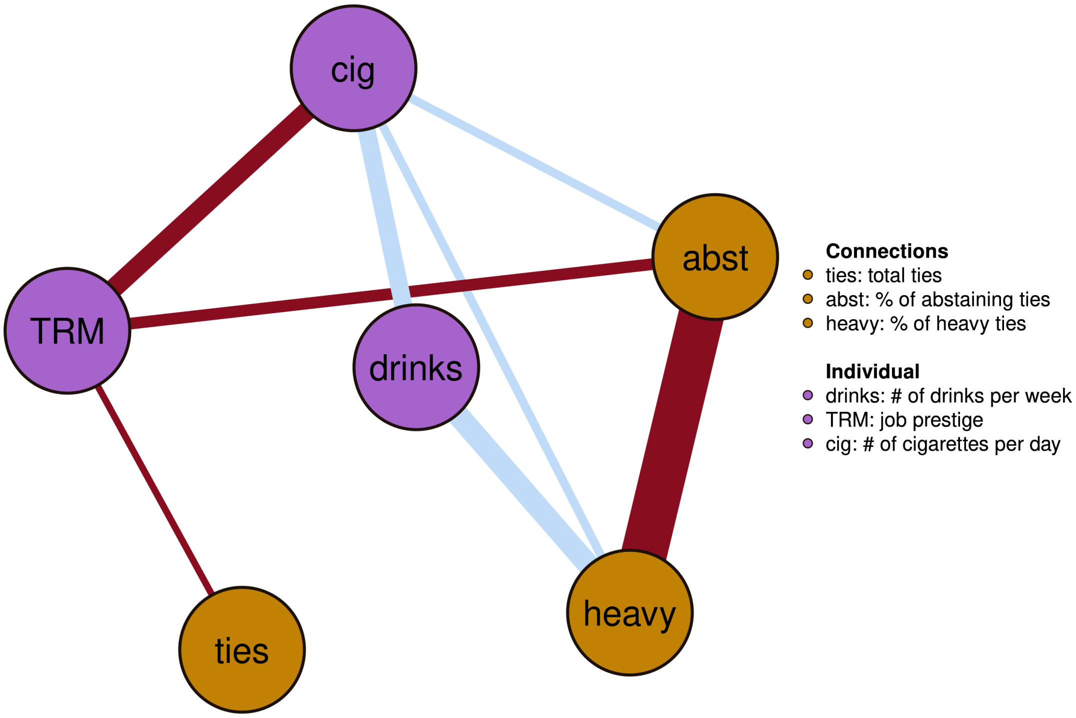

Figure S2: Fixed-effect between associations within the same time window of solely the Offspring Cohort. Blue are positive and red are negative associations, the width of the edge represents the strength. Node color indicates personal (purple) or social (gold) variable.

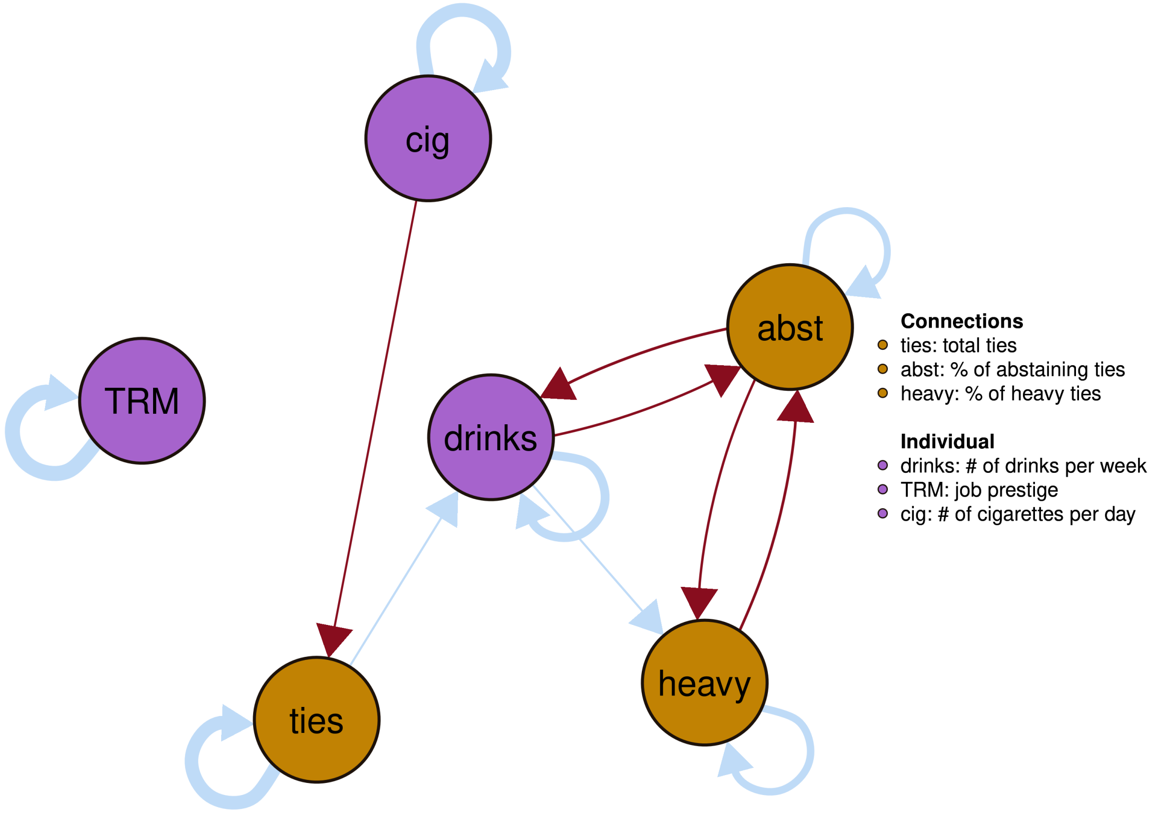

Figure S3: Fixed effect temporal associations of solely the Offspring Cohort. Blue are positive and red are negative associations. The thickness of the arrow indicates the strength of the association. Node color indicates personal (purple) or social (gold) drinking behaviour variables. Notably, unlike in the full population, the reciprocal edge between percentage of heavy drinking ties and number of drinks is not present.
